# Long-Term Results at 10 Years of Pouch Resizing for Roux-en-Y Gastric Bypass Failure

**DOI:** 10.3390/nu14194035

**Published:** 2022-09-28

**Authors:** Céline Drai, Andrea Chierici, Luigi Schiavo, Tagleb S. Mazahreh, Anne-Sophie Schneck, Antonio Iannelli

**Affiliations:** 1 Department of Digestive Surgery and Hepatic Transplantation, Centre Hospitalier Universitaire de Nice, Hôpital Archet 2, 06202 Nice, France; 2 Faculty of Medicine, University Côte d’Azur, 06202 Nice, France; 3 Department of Medicine, Surgery and Dentistry, University of Salerno, 84081 Baronissi, Italy; 4 Department of General Surgery and Urology, Faculty of Medicine, Jordan University of Science & Technology, Irbid 22110, Jordan; 5 Department of Digestive Surgery, Centre Hospitalier Universitaire de Guadeloupe, Pointe à Pitre 97159, Guadeloupe; 6 Inserm, U1065, Team 8 “Hepatic Complications of Obesity and Alcohol”, C3M Bâtiment Universitaire ARCHIMED, 06204 Nice, France

**Keywords:** Roux-en-Y gastric bypass, weight loss failure, weight regain, gastric pouch resizing

## Abstract

Background: Roux-en-Y gastric bypass (RYGB) is currently one of the most performed bariatric procedures and it is associated with rapid weight loss. However, weight loss failure and weight regain after RYGB occurs in approximately 30% and 3–5% of patients, respectively, and represent a serious issue. RYGB pouch resizing is a surgical option that may be offered to selected patients with RYGB failure. The aim of this study is to assess long-term results of pouch resizing for RYGB failure. Materials and Methods: From February 2009 to November 2011, 20 consecutive patients underwent gastric pouch resizing for RYGB failure in our tertiary bariatric center. The primary outcome was the rate of failure (%EWL < 50% with at least one metabolic comorbidity) after at least 10 years from pouch resizing. Gastroesophageal Reflux Disease (GERD) was also assessed. Results: Twenty patients (18 women (90%)) were included and seventeen (85%) joined the study. The failure rate of pouch resizing was 47%. Mean %EWL and mean BMI were 47%, and 35.1 kg/m^2^, respectively. Some of the persistent co-morbidities further improved or resolved after pouch resizing. Seven patients (41%) presented GERD requiring daily PPI with a significantly lower GERD-HQRL questionnaire score after pouch resizing (*p* < 0.001). Conclusion: Pouch resizing after RYGB results in a failure rate of 47% at the 10-year follow-up while the resolution of comorbidities is maintained over time despite a significant weight regain.

## 1. Introduction

Bariatric surgery (BS) has developed enormously over the last two decades in concomitance with the obesity epidemic [1,2]. Prospective studies have shown that BS is associated with significant and sustained weight loss, remission or improvement of obesity-associated medical problems as well as reduced mortality, stroke, and cancer risk [2]. Several longitudinal studies have shown the short- and medium-term efficacy of Roux-en-Y gastric bypass (RYGB) and these results were confirmed by meta-analyses [3]. However, very few data are available concerning the results of the RYGB beyond 10 years. Weight loss failure or regain are common in the long-term (30% and 3–5%, respectively) [4,5] and they are often associated with the recurrence or the development of new obesity-associated medical problems [4,6]. The definition of failure is not consensual in the literature; however, most of the authors agree on a loss of excess weight (%EWL) of less than or equal to 50%, associated with the recurrence of one or more obesity-related medical problems [3,6]. The prevalence of bariatric surgery failure is largely unclear, mainly because an all-encompassing, unambiguous definition is lacking. Adams et al. reported in a controlled study that 30% out of 418 participants with a RYGB showed <20% of total body weight loss at 12 years from surgery [7]. A recent study based on the Scandinavian Obesity Surgery Registry showed that the prevalence of RYGB failure 5 years after surgery was 23% [8]. These figures indicate that RYGB failure at mid- and long-term is an important issue in bariatric surgery. The reasons for inadequate weight loss or weight regain after RYGB relies on many isolated or concomitant factors: gastric pouch dilatation, insufficiently small gastric pouch (>5 cm), wide gastro-jejunal anastomosis (>1–1.5 cm), secondarily enlarged gastro-jejunal anastomosis, postoperative complications such as gastric fistula, short biliopancreatic limb, and hyperphagic behavior [9].

Gastric pouch resizing (GPR) is a surgical option that may be offered to selected patients with RYGB failure. The principle is to restore restriction by decreasing the capacity of the stomach. Given the high number of RYGB performed yearly worldwide, the fact that this procedure was introduced more than four decades ago now, and the rate of failure that increases with time from the index surgery, bariatric surgeons have investigated the possibility of restoring restriction. However, there is conflicting evidence in the literature regarding the efficacy and the safety of GPR [10,11]. Furthermore, the results of this surgical option have never been reported in the long-term. 

As we previously reported the results of GPR in a series of 20 consecutive patients [12], we aimed in this study, to investigate the rate of failure in this same cohort of individuals with a minimum follow-up of 10 years. 

## 2. Materials and Methods

### 2.1. Study Design

This is a single-center longitudinal cohort study. The short-term results of this cohort were published previously and this study replicates the long-term follow-up of this longitudinal cohort [12]. Data were retrieved retrospectively from a prospective held database in our tertiary referral bariatric center and updated with a minimum follow-up of 10 years. This study was declared to the *Commission Nationale Informatique et Liberté (CNIL 6009701)*. 

From February 2009 to November 2011, 20 consecutive patients underwent GPR for RYGB failure. All patients in our study had the RYGB performed with a 150 cm long alimentary limb and a 50 cm long biliopancreatic limb and a 10–12 mm hand-sewn gastro-jejunostomy. The indications for GPR were progressive weight regain with recurrence of at least one comorbidity in 13 patients (65%) and insufficient weight loss (%EWL < 50%) in seven patients (35%). Both RYGB and GPR were performed laparoscopically in all patients. All data were collected prospectively. 

All patients were examined in the outpatient’s clinic between July and September 2021 after a 10-year follow-up from GPR, to evaluate weight loss and obesity medical-associated problems evolution. 

The primary endpoint was the long-term failure rate of GPR, defined as a composite endpoint including <50% of excess weight loss (%EWL), and/or the presence of at least one obesity medical-associated problem (persistent, recurrent after remission or improvement, or de novo) among type 2 diabetes (T2D), dyslipidemia, hypertension (HT); osteoarthritis (OA), obstructive sleep apnea syndrome (OSAS). The secondary endpoints were the assessment of the evolution of the associated medical problems (T2D; HT; OSAS, OA, GERD). The presence of associated medical problems was classified as recurrence (in patients for whom a remission had been proven during follow-up) or de novo onset (in patients not presenting the comorbidity before surgery).

Patients’ informed consent was obtained in all cases. To investigate, Gastroesophageal Reflux Disease (GERD) patients were also asked to complete the Gastroesophageal Reflux Disease (GERD-HRQL) Health-Related Quality of Life Scale (Appendix A) which focuses on pyrosis-like symptoms, dysphagia, effects of medication, and consequences of GERD on patients’ daily life [13]. All patients who regained weight after RYGB underwent psychiatric and nutritional assessment prior to re-operation. Data on %EWL after GPR were calculated from the preoperative weight at the time of RYGB. The method to realize the volumetric assessment of the gastric pouch was reported previously [12]. 

### 2.2. Measure of Weight Loss and Obesity-Associated Medical Problems Definitions

The ideal weight in kilograms was calculated according to the following formula: 50 + 2.3 × ((height in cm/2.54) − 60) for men and 45.5 + 2.3 × ((height in cm/2.54) − 60) for women. Excess weight was calculated as follows: preoperative weight − ideal weight; %EWL was calculated according to the following formula (preoperative weight − postoperative weight)/(preoperative weight − ideal weight) × 100.

T2D was defined as a circulating glucose level above 1.25 g/L or glycated hemoglobin above 7%. A decrease in the number of medications associated with a circulating glucose level of less than 1 g/L or glycated hemoglobin of less than 6.5% or a decrease in the number of diabetes-related medications associated with a circulating glucose level between one and 1.25 g/L defined T2D resolution or improvement. Data on circulating glucose levels were checked on blood tests when available or through the endocrinologist or family doctor in charge of patients. HT was defined as a systolic pressure greater than 130 mm of mercury (mmHg) or a diastolic pressure greater than 85 mmHg or treatment associated with hypertension. Resolution or improvement of HT was defined as a decrease in the number of medications associated with a systolic pressure below 130 mmHg or a diastolic pressure below 85 mmHg. The presence of OSAS was quantified by mandatory preoperative polysomnography before RYGB, and its resolution was confirmed by turning off the positive pressure machine postoperatively. OA-related pain was investigated through patients’ anamnesis, pain regression and/or reduction in OA-related medications was taken into account to evaluate OA evolution. The presence of GERD was determined through the GERD-HQRL questionnaire [13]. 

### 2.3. Surgical Technique

All patients underwent GPR without redo of the gastro-jejunostomy. The entire gastric pouch was released by exposing the left pillar of the diaphragm. The gastro-jejunostomy anastomosis was measured between 12 and 14 mm in diameter by preoperative endoscopy in all cases. The gastrectomy was performed by vertical stapling of the pouch (Endo-GIA Black loads—Medtronic, Fridley, MN, USA) reinforced with a bioabsorbable membrane (GORE^®®^ SEAMGUARD^®®^) over a 34 Fr calibration tube. A leak test of the staples was systematically performed intraoperatively. A drain was left in place at the end of each procedure. 

### 2.4. Statistical Analysis

The results are presented as means with standard deviations and medians with interquartile ranges when suitable. Statistics were performed with the R software [14]. For the comparison of weight loss and BMI, the Mann–Whitney test was used, calculated with reference to the preoperative weight before RYGB. For GERD and GERD-HQRL score, the Student’s *t*-test was used to compare means. 

## 3. Results

### 3.1. Study Population

Patients’ characteristics at baseline are reported in Table 1. Twenty patients including 18 women (90%) were selected and 17 (85%) were available for follow-up and consented to participate in the present study. The mean follow-up was 136.9 months. Before the index RYGB the initial BMI was 45 kg/m^2^ (42.6–49.6) and the initial mean age was 43 ± 10 years. The time from initial surgery to GPR was 49 ± 24 months. The lowest BMI achieved after RYGB was 30.9 kg/m^2^ (27.2–35.8). BMI and %EWL at the time of GPR were 33.3 kg/m^2^ (31.8–37.6) and 44.9% (32.5–52.4), respectively. 

### 3.2. Primary Outcome 

The failure rate of GPR was 47% at 10 years. Eight patients had failed the sequence of RYGB and GPR: seven (41%) had a %EWL < 50% and one (6%) patient had persistent OSAS despite a %EWL loss of 52%. Three patients did not complete the 10 years follow-up. At 10 years, median %EWL and median BMI were 69.2% (60.8–77.5), and 29.5 kg/m^2^ (26.9–31.4), respectively. The minimum BMI reached one year after GPR was 31 kg/m^2^ (26.9–34.7) and corresponded very closely to the minimum BMI reached after the RYGB. Overall, the two procedures allowed patients to lose 51.5% of their excess BMI at 10 years, with three patients (15%) lost at follow-up. Of the seven patients with a %EWL < 50% at 10 years, five had at least one persistent medical-associated problem, and four (24%) had GERD on long-term proton pump inhibitor. Among the 10 patients with a %EWL > 50% at 10 years, one (6%) patient had OSAS needing positive pression therapy despite a %EWL of 52% and three (18%) patients had GERD on a long-term proton pump inhibitor. 

### 3.3. Evolution of Obesity-Related Medical Problems

Table 2 shows the evolution of obesity-related comorbidities over the study period indicating that GPR resulted in the further resolution and/or improvement of some of the obesity-related medical problems and this was maintained after the 10-year follow-up. Eight (40%) patients had pre-operative disabling OA. At the time of GPR, OA improved for three patients (15%) and two patients (10%) were pain free. At last follow-up, six patients (35%) had symptoms: three (18%) were less symptomatic and described a significant reduction in pain and three (18%) had persistent pain. 

RYGB, Roux en Y Gastric Bypass; GPR, Gastric Pouch Resizing; HT, Hypertension; OSAS, obstructive sleep apnea syndrome.

At the 10-year follow-up from GPR, seven patients (41%) presented GERD requiring daily proton-pump inhibitor but the GERD-HQRL questionnaire score was significantly lower than before GPR (Table 3). Finally, two patients (12%) presented with multiple episodes of vomiting (3–5 times/week) despite fractioned food intake and dietician follow-up.

## 4. Discussion

This study shows that GPR after RYGB results in a failure rate, defined as %EWL < 50% and/or persistence, recurrence, or de novo development of at least one obesity-related comorbidity of 47% at the 10-year follow-up. Moreover, we found that the resolution of comorbidities is maintained over time despite a significant weight regain. Hamdi et al. and, later, Borbély et al., reported the results of 25 and 26 patients, respectively, after GPR [10,15]. Both the research focused on the early- and mid-term results (24 and 48 months of follow-up respectively) showing contrasting results although the populations and the indications for surgery were apparently superposable. Hamdi et al. reported efficacy of GPR until a 1-year follow-up with subsequent weight regain after 2 years from surgery, while Borbély et al. found more encouraging results with a mean BMI of 33.8 kg/m^2^ after a 4-year follow-up in their population. This important difference can be due to the difference in the criteria chosen reported by the two authors to perform GPR. Complication rates were between 8 and 15% depending on the series. In addition, a study by Felsenreich et al. in 2019 [16] reported 69% ± 32.5% %EWL over a mean follow-up of 43 months after GPR in a series of 40 patients. In this study, four different procedures of RYGB revision for weight regain were applied. The results obtained with GPR were comparable to those obtained with other procedures including GP banding, GP banding plus resizing, and common limb shortening. However, the authors showed that the addition of a band around the GP resulted in dysphagia and common limb shortening was associated with a considerable risk of malnutrition.

Only few studies reported a follow-up beyond 5 years and most patients were lost, even in studies with a shorter follow-up. 

The development of GERD following the RYGB that is considered as an “anti-reflux” procedure may seem nonsensical, but it is an important point as seven patients (41%) in our series reported the need for daily proton-pump inhibitor, although the GERD HQRL score decreased significantly after GPR at the 10-year follow-up. Several hypotheses can be put forward for the development or the persistence of GERD including the presence of hypersensitive esophagus, functional impairment of the lower esophageal sphincter in the presence of a pouch which is narrower than the esophagus and may then act as a mechanical obstacle to esophageal emptying. In the absence of endoscopic, manometric, and pH metric studies, it is difficult to establish the mechanisms responsible for reflux in these patients. However, a recent study by Dupree et al. showed that the rate of reflux and proton-pump inhibitors at long-term use is high after RYGB [17]. In 33,847 RYGB patients, more than 60% of patients with preoperative GERD symptoms were improved after RYGB, 30% felt no difference, and 10% experienced worsening of their symptoms. As a matter of fact, GERD is a well-known issue associated with bariatric surgery and its evolution should always be described in research analyzing the efficacy of a bariatric procedure which is lacking in current literature on GPR.

Face to weight loss failure or regain after RYGB, other options have been reported including the lengthening of the alimentary and or biliopancreatic limb with or without the resizing of the gastro-jejunostomy in adjunction to GPR with variable results and in a short follow-up series.

Wijngaarden et al. [18] focused their attention on comparing two different surgical technique of stomach resizing with or without resection and creation of a new gastro-jejuno anastomosis. They showed no significant difference when the redo of the gastro-jejunostomy was performed with GPR in terms of weight loss and safety which were equivalent in the short term (2 years).

The most common alternative to GPR in the case of weight loss failure or weight regain is represented by common limb shortening. The first case series of 29 patients undergoing this procedure after RYGB failure is the one reported by Rawlins et al. [19] with excellent results in terms of sustained weight loss after revision (a mean 68.8%EWL and 31.5 Kg/m^2^ of BMI after a 5-year follow-up). In addition, Krajlevic et al. [20] also described the effects of the biliopancreatic limb lengthening in 28 patients with failure of RYGB with a 50 cm long biliopancreatic limb; a significant improvement in weight loss measures was already seen after a 1-year follow-up and more than 50%EWL was obtained at 5-years from procedure but only seven patients completed the follow-up. Although these two research focus on the promising long-term results of common limb shortening, an increased incidence of postoperative malnutrition (24% and 21.4%, respectively) with the need to proceed to reoperation and reversal is the major concern for both studies. As a matter of fact, alimentary, biliopancreatic, and common limbs length are part of an unsteady balance between the risk of insufficient weight loss and the development of nutritional deficiencies on the other hand. Finding the “perfect length” reaching the exact balance that leads to an increased loss of weight without the risk of proteic malnutrition is a risky strategy as the latter is a complex clinical situation associated with a high mortality [21]. Considering this specific subject, what is reported in a recent case series by van der Burgh et al. [22] is very suggestive. Forty-seven patients with insufficient weight results after a RYGB with a 100–150 cm alimentary limb and a 75 cm biliopancreatic limb underwent distalization of the entero-entero anastomosis to produce a 100 cm common channel. Despite the fact that 24 patients succeeded in achieving adequate weight loss after 34 months of follow-up, an impressive 89% of patients developed nutritional deficiencies at different extents even with a correct vitamins and minerals supplementation. In addition, 22 patients declared to have a reduced quality of life due to debilitating defecation patterns after surgical revision. In the whole cohort, 10 patients needed reoperation with five having common channel proximalization to 250 cm with the Authors concluding that a >200 cm common channel and a close postoperative patient monitoring are mandatory.

To investigate more precisely how slipping from inadequate weight loss to malnutrition is a matter of centimeters, Ghiassi et al. [23] published their experience on common limb shortening for RYGB failure with different common limb lengths. They reported the results of weight loss and nutritional deficiencies in 11 patients who had distalization of entero-enterostomy to obtain a 150–200 cm common limb and in other 85 subsequent patients who had a 300–350 cm common limb. Three-years BMI and %EWL were 32.2 kg/m^2^ and 65.7% attesting encouraging mid-term results on weight loss of RYGB revision; however, seven patients with a 250–300 cm common limb experienced malnutrition and diarrhea requiring reoperation while in the 400–450 cm common limb group of patients there was no need of reoperation for malnutrition. Moreover, a significant reduction in hemoglobin, serum protein, and calcium from baseline to three years after RYGB revision was found also in the longer common limb group. 

Another study by Ortega-Serrano et al. [24] on 23 patients with weight regain after RYGB undergoing common limb shortening with a mean follow-up of 35 months, showed that the shortening of the common limb (100–150 cm) permitted to regain the same minimum BMI as after RYGB with only four patients presenting with iron or vitamin D deficiency at last follow-up. 

Little cohorts with heterogenous indications, different surgical techniques and short follow-up are probably responsible for these differing inter-research results but malnutrition represents a common issue.

Another surgical weapon to obtain further weight loss in case of RYGB failure which has been lately described consists in reducing the gastric size and outlet by performing a laparoscopic gastric banding. Uittenbogaart et al. [25] published a single-center cohort of 44 patients undergoing this procedure. These patients had widely different results in terms of weight loss with a reported 17.6 ± 28.3 %EWL; moreover, patients with previous weight regain after RYGB were reported to have better results than those who experienced insufficient weight loss. Again, a short follow-up with many patients missing their routine postoperative visits makes generalization of these findings inappropriate.

In the end, a few case series of conversional surgery from a failed RYGB to a different already well-described bariatric reconstruction have been published lately with interesting insights on the effects on weight loss and the associated complications. 

De Luca et al. [26] detailed the results of performing a Single-Anastomosis Jejuno-Ileal bypass after a failed RYGB in 31 patients. Mean BMI, %EWL, and %TWL progressively decreased through all the time-points of their 48-month follow-up highlighting promising effects of bariatric revisional surgery with a moderate complication rate (37.2% early postoperative morbidity and 50% late morbidity) consisting preeminently in Clavien-Dindo I and II grade complications. Unfortunately, no data were reported concerning the nutritional status of these patients which can be widely affected as the conversion to Single-Anastomosis Jejuno-Ileal bypass consists in an important reduction in length of the common channel with a pronounced malabsorptive effect.

Similarly, Topart et al. [27] and Surve et al. [28] faced this issue proposing conversional bariatric surgery to Biliopancreatic Diversion with Duodenal Switch to patients who failed to achieve adequate and long-lasting weight loss after RYGB. In both cases, the authors described impressive weight loss results throughout the follow-up. Surve et al., found 29.2%EWL and 56.4%TWL compared with the last measure before conversion after a 24-month follow-up in their 32 patients case series; Topart et al. described a 73.5%EWL and a 37.6%TWL from the initial weight before RYGB in 14 patients after a mean a 25.8-month follow-up. Both the authors also reported data regarding post-conversion nutritional outcomes. In the study by Topart et al. there were isolated cases of hypovitaminosis D and A, hypoalbuminemia, and diarrhea at the last follow-up.

A meta-analysis by Kermansaravi et al. [9] evaluating the efficacy of different procedures after RYGB failure shows that the best weight loss results are obtained with conversion to Duodenal Switch or Single-Anastomosis Jejuno-Ileal bypass, followed by shortening of the common channel. Although this research lacks a between procedures comparison of early and late postoperative complications, reports from each study highlights an increased rate of late complications when a distalization of the entero-enteric anastomosis is performed, notably nutritional deficiencies of different severity.

Whatever strategy is chosen, it should be kept in mind that combining an increased restriction by GPR with increased malabsorption is associated with a significant increase in the risk of proteic malnutrition which should be avoided [29,30]. 

The recent results obtained with the incretins analogues in individuals with a BMI at 27 and at least one obesity-related complication and those with a BMI > 30 are of great interest and may revolutionize the field of bariatric surgery in the foreseeable future [31]. However, transposing the results of these studies on incretins analogues to the selected population of individuals experiencing bariatric surgery failure should be performed with care as additional weight loss is more difficult to obtain in this setting. However, although no solid data are available so far in the setting of RYGB failure, undergoing incretins analogues treatment especially beyond one-year follow-up, it may be speculated that these drugs play a major role in the treatment algorithm for these individuals.

The main strengths of this study are the length of follow-up which has not been reported so far in the literature, the standardization of the surgical technique, and the small number of patients lost to follow-up. On the other hand, the small number of patients included make the extrapolation of the results on a larger scale difficult, even if the success rate is calculated at 53%. Indeed, these data should be confirmed in a larger, multicenter study with the same long-term follow-up. Moreover, the measurement of the gastric pouch volume was performed before accurate computed tomography methods were available, except for the last three patients.

## 5. Conclusions

GPR may be an attractive option for failed weight loss in selected patients with a dilated gastric pouch. The efficacy of this option is proven in the short term and this study shows that 53% of patients have good results in the long term when considering weight loss. Moreover, a substantial improvement in GERD-related symptoms and in obesity-associated medical problems has been highlighted after GPR. Other solutions to RYGB failure have been proposed with promising results in terms of weight loss but elevated rates of postoperative complications and nutritional deficiencies. Larger, multicentric, long-term follow-up studies should be performed to confirm these results.

## Figures and Tables

**Table 1:** Patients’ characteristics at baseline.

Number of Patients	20
Age before RYGB, years	43 ± 10
Sex, men	2 (10)
Weight before RYGB, kg	118 (110–130.5)
BMI before RYGB	45 (42.6–49.6)
Minimum BMI after RYGB	30.9 (27.2–35.8)
Delay between RYGB and GPR, m	49 ± 24
BMI before PR	33.3 (31.8–37.6)

Categorical variables are expressed as *n* (%); continuous variables are expressed as mean ± SD or median (IQR).

**Table 2:** Evolution of obesity-associated medical problems.

Associated Medical Problems	Before RYGB	Before GPR	After GPR *
HT	7 (35%)	5 (25%)	4 (24%)
Improved HT		1 (5%)	1 (6%)
Type 2 diabetes	5 (25%)	2 (20%)	0
Improved diabetes		2 (20%)	1 (6%)
OSAS	5 (25%)	2 (10%)	0
Improved OSAS		-	1 (6%)
Disabling osteoarthritis	8 (40%)	3 (15%)	3 (18%)
Improved osteoarthritis		3 (15%)	3 (18%)

Categorical variables are expressed as *n* (%). * Time point corresponds to the last available follow-up ≥10 years after GPR.

**Table 3:** Prevalence of Gastroesophageal Reflux, GERD HQRL Quality of Life Questionnaire.

GERD HQRL	*n* (%)	*p*
Patients with GERD	7 (41%)	
Daily PPI intake	7 (41%)	
* GERD HQRL score	23 ± 2	<0.001
** GERD HQRL score	15 ± 1	
Are you satisfied with current results of RYGB?		
yes	9 (53%)	
no	8 (47%)	

Categorical variables are expressed as *n* (%); continuous variables are expressed as means (SD). * Mean GERD HQRL score is calculated for GERD suffering patients only before GPR; ** Mean GERD HQRL score is calculated for GERD suffering patients only after 10-year follow-up from GPR.

## Data Availability

The data presented in this study are available on request from the corresponding author. The data are not publicly available due to privacy reasons.

## Appendix A. GERD-HQRL Questionnaire Scale

- 0 = no symptoms
1. 1 = noticeable but not bothersome symptoms.
2. 2 = noticeable and annoying symptoms, but not every day.
3. 3 = Symptoms that are annoying every day.
4. 4 = Symptoms that affect daily activities.
5. 5 = Symptoms are incapacitating (unable to perform daily activities).

**Questions**

1. How severe is your heartburn?  1 2 3 4 5
2. Do you have heartburn while lying down?  1 2 3 4 5
3. Do you have heartburn while standing? 1 2 3 4 5
4. Do you have heartburn after meals?  1 2 3 4 5
5. Does heartburn change your diet?  1 2 3 4 5
6. Does heartburn wake you up at night?  1 2 3 4 5
7. Do you have difficulty swallowing? 1 2 3 4 5
8. Does it hurt when you swallow?  1 2 3 4 5
9. Do you feel gassy or bloated?  1 2 3 4 5
10. If you are taking medication, does this affect your daily life?  1 2 3 4 5
11. Are you satisfied with your quality of life?  Satisfied—neutral—dissatisfied
